# Subcutaneous extension of perigraft seroma after thoracic aortic surgery — report of three cases

**DOI:** 10.1186/s44215-022-00017-y

**Published:** 2023-03-17

**Authors:** Tsutomu Doita, Taro Yamasumi, Yuki Nakamura, Haruhiko Kondoh

**Affiliations:** Department of Cardiovascular Surgery, Japan Organization of Occupational Health and Safety, Osaka Rosai Hospital, Sakai, Osaka 591-8025 Japan

**Keywords:** Perigraft seroma, Thoracic aorta, Total arch replacement, Subcutaneous tumor

## Abstract

**Background:**

Subcutaneous extension of a perigraft seroma following thoracic aortic surgery is an infrequently encountered complication. For treatment, it is necessary to first exclude the possibility of perigraft fluid collection secondary to infection, a pseudoaneurysm, or impending rupture. However, it is difficult to diagnose collected fluid as indicative of a perigraft seroma, and there is also no standardized treatment for this condition.

**Case presentation:**

Three patients who had undergone graft replacement of a thoracic aorta with arch reconstruction were referred to our department for findings of a subcutaneous tumor. In each, computed tomography showed perigraft fluid accumulation in the posterior sternum. In one, a subcutaneous tumor had appeared in the center area of the median sternotomy, 4 months after the operation. It was considered that a wound infection was the likely cause; thus, negative pressure wound therapy was started after incisional drainage and debridement. However, a fever developed 14 days after debridement, resulting in mediastinitis, the latter of which was assumed to be caused by a retrograde infection of the incisional wound. In the other two cases, a subcutaneous tumor appeared in the center of the median area of the sternotomy at 3 and 4 months, respectively, after the operation. In both, the tumor was treated with continuous drainage by aspiration with negative pressure, and cultures of drainage samples were negative for infection. Neither patient had a fever during hospitalization, and fluid collection disappeared during follow-up. In one of the latter two cases, a subcutaneous tumor formed again 3 months after drainage, which was treated in the same manner and subsequently disappeared. Thereafter, there was no recurrence noted at the 3-year follow-up examination.

**Conclusion:**

A perigraft seroma following thoracic aortic surgery, an infrequently encountered complication, has an appearance is similar to a subdermal tumor. Continuous aspiration drainage is a useful option for patients with no fever and negative for infection in drainage culture results.

## Background

Few reports of treatment for a perigraft seroma that developed after thoracic aortic surgery have been presented in literature. Identification in clinical settings is difficult, and diagnosis is generally determined following exclusion of infection, pseudoaneurysm, and other conditions. When a perigraft seroma is present but does not compress any organs or cause symptoms, it should be examined. However, perigraft fluid collection secondary to an infection or development of a pseudoaneurysm after thoracic aorta graft replacement can be lethal. Thus, careful management for such fluid collection is necessary. Herein, we report three cases of subcutaneous extension of a perigraft seroma following thoracic aortic surgery.

## Case presentations

### Case 1

**Fig. 1:**
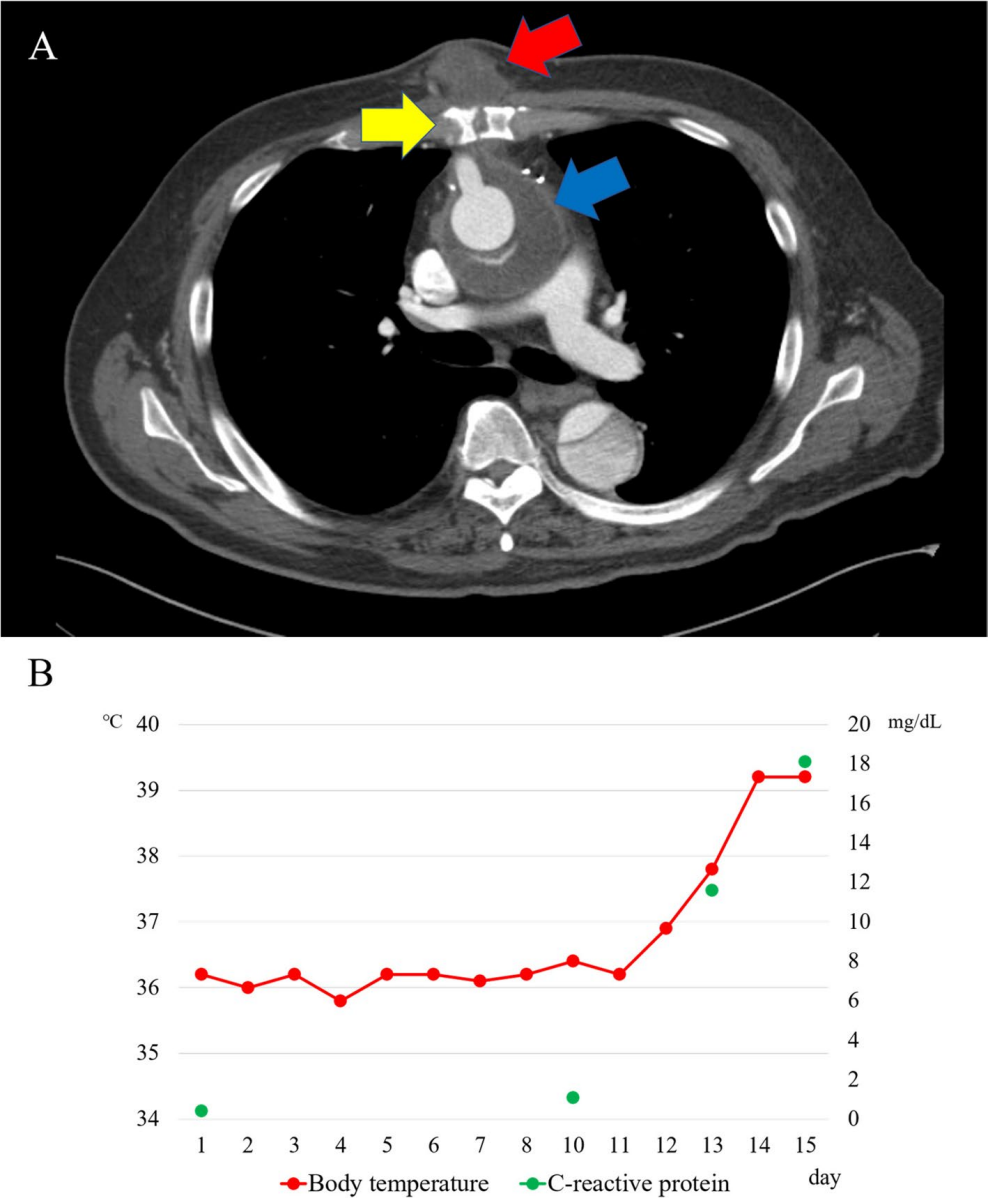
**A** Enhanced CT findings in case 1. Enhanced CT revealed a subcutaneous low-density mass (red arrow) sized 33 × 32 mm in the center of the median sternotomy, continuous with a 25-mm-wide area of fluid collection (blue arrow) around the ascending aortic graft with no extravasation of contrast media observed. The gap of the closed sternum was also visualized (yellow arrow). **B** Chart for case 1 patient body temperature and C-reactive protein level. Shown is the chart of body temperature (red point) and C-reactive protein levels (green point) recording during hospitalization. On day 12 after admission, a fever developed, and the creative protein level was drastically elevated to 11.2 mg/dL

A 68-year-old man with a history of smoking, atrial fibrillation, and lacunar infarction was referred to us for evaluation of chest pain. Computed tomography (CT) revealed a type A aortic dissection causing upper-right extremity malperfusion. He previously underwent emergency surgery for ascending aorta replacement with a right subclavian artery bypass procedure using a one-branch polyester knitted Dacron graft (26 × 10 mm Triplex, Terumo Corporation, Tokyo, Japan). At a routine visit 4 months after the operation, a subcutaneous tumor approximately 3 cm in diameter without reddening appeared in the center of the median sternotomy area. Enhanced CT showed a subcutaneous low-density mass sized 33 × 32 mm, with a continuous 25-mm-wide area of fluid collection around the ascending aortic graft and no extravasation of contrast media (Fig. 1A). A physical examination showed a normal body temperature of 36.4 °C, while blood tests revealed a white blood cell (WBC) count of 5800/μL and C-reactive protein (CRP) level of 0.69 mg/dL. Even though body temperature was normal, and there was no evidence of elevated inflammatory markers or infectious signs in the wound area, the possibility of infection could not be excluded, and incisional drainage of the subcutaneous tumor was performed. The wound culture was negative; thus, it was treated with cleansing and negative pressure wound therapy (NPWT).

Twelve days after admission, the patient developed a fever, with WBC count and CRP level drastically elevated to 11,600/μL and 11.2 mg/dL, respectively (Fig. 1B). Another wound culture was performed, which revealed *Staphylococcus aureus*. Additionally, CT findings showed perigraft gas formation; thus, graft infection and mediastinitis were diagnosed, and a redo sternotomy, cleansing, and debridement around the prosthetic graft and an omentopexy were performed. The postoperative course was uneventful, and the patient was discharged after wound healing was confirmed. It is assumed that the graft infection and mediastinitis were caused by a retrograde infection of the incised subcutaneous tumor, which was connected following perigraft fluid collection through the gap of the closed sternum.

### Case 2

**Fig. 2:**
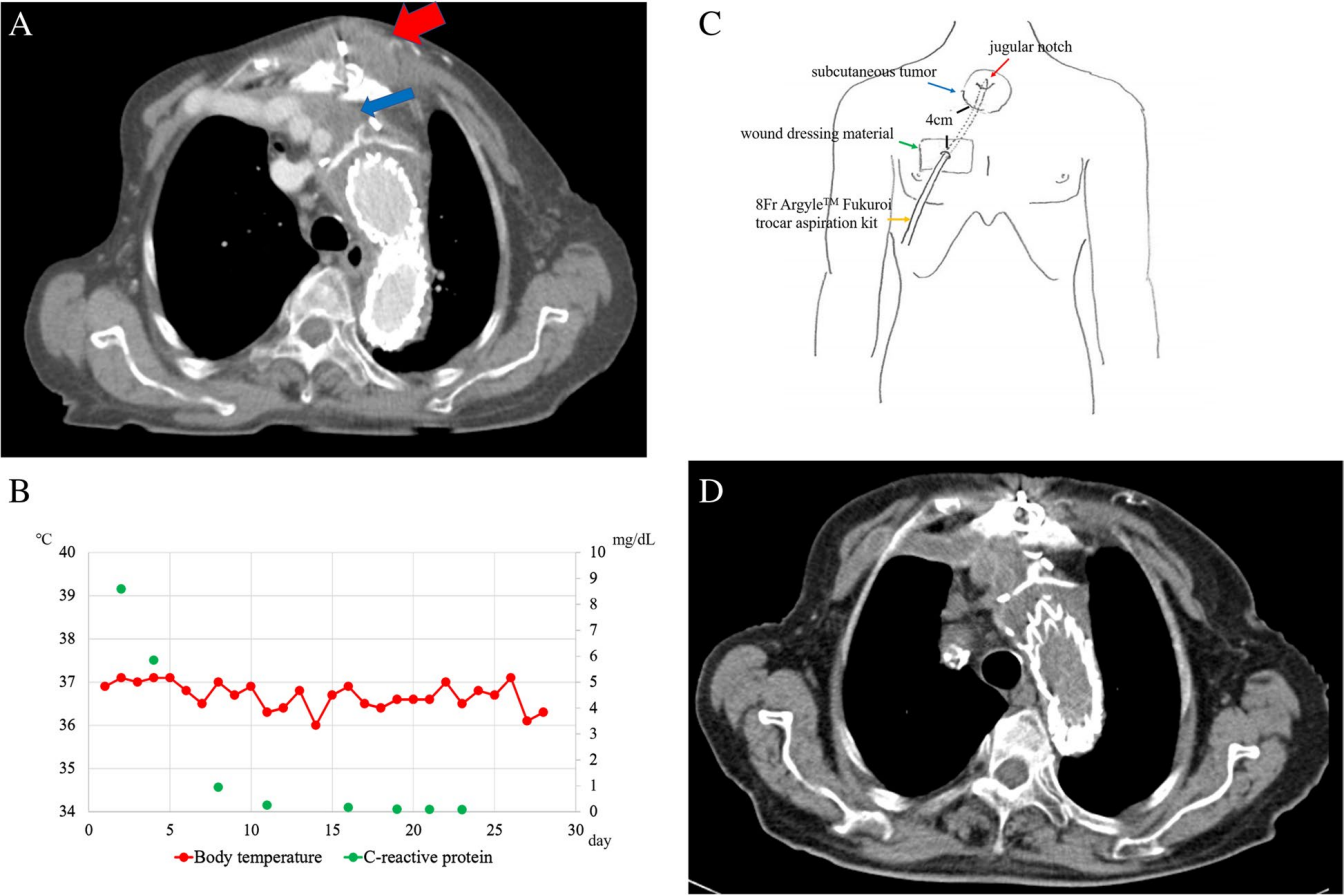
**A** Enhanced CT findings in case 2. Enhanced CT revealed a subcutaneous low-density mass (red arrow) sized 21 × 43 mm in the center of the median sternotomy, continuous with a 19-mm-wide area of fluid collection (blue arrow) around the ascending aortic graft with no extravasation of contrast media observed. **B** Chart for recording case 2 patient body temperature and C-reactive protein level. Shown is the chart of body temperature (red point) and C-reactive protein levels (green point) recorded during hospitalization. No fever was noted during hospitalization, while the C-reactive protein level showed a rapid decrease just after starting antibiotic therapy for pneumonia. **C** Schema of continuous aspiration drainage catheter treatment. The patient was treated with continuous aspiration drainage under negative pressure using a small-bore aspiration catheter (8Fr Argyle™ Fukuroi trocar aspiration kit, Cardinal Health, Dublin, Ohio) inserted through a subcutaneous point 4 cm from the tumor. A catheter insertion site kept clean by covering with a wound dressing material, in order to prevent from a retrograde infection. **D** CT findings 1 month after treatment with aspiration drainage. The subcutaneous tumor had disappeared, and no perigraft fluid was collected.

An 83-year-old female with a history of cerebral infarction underwent first-stage total arch replacement using a four-branch polyester knitted Dacron graft (28 × 9 × 9 × 11 mm, Triplex, Terumo Corporation, Tokyo, Japan) with an elephant trunk (ET) for an ascending arch aortic aneurysm with chronic dissection, followed by second-stage endovascular ET completion. A subcutaneous tumor approximately 2 cm in diameter without reddening appeared in the center of the median sternotomy area at 7 months after the first procedure. Enhanced CT showed pneumonia and a subcutaneous low-density mass sized 21 × 43 mm in the center of the sternotomy, with a continuous 19-mm-wide area of fluid collection around the ascending aortic graft and no extravasation of contrast media observed (Fig. 2A). A physical examination showed a normal body temperature of 36.9 °C, with a WBC count of 6600/μL, and CRP level of 8.59 mg/dL noted in blood tests. CRP was elevated due to pneumonia and that was treated with antibiotic therapy, after which the level rapidly returned to within normal limits (Fig. 2B). Exploratory aspiration of the tumor was performed, and a culture test was negative. There was no evidence of wound infection or graft infection, including normal body temperature, negative aspiration culture, and no infectious signs in the area of the wound. Therefore, a perigraft seroma was suspected.

The tumor was treated with continuous aspiration drainage under negative pressure using a small-bore aspiration catheter (8Fr Argyle™ Fukuroi trocar aspiration kit, Cardinal Health, Dublin, Ohio), inserted through a subcutaneous point 4 cm from the tumor, and use of a continuous negative pressure system (J-VAC®, Ethicon™, Somerville, NJ, USA) (Fig. 2C). Cultures of drainage remained negative, and there was no fever during the time of admission. WBC count and CRP level did not elevate, and the tumor was reduced in size; thus, removal of the drain tube was performed at 18 days after insertion. Thereafter, there was no subcutaneous tumor recurrence and perigraft fluid collection disappeared after discharge (Fig. 2D).

### Case 3

**Fig. 3:**
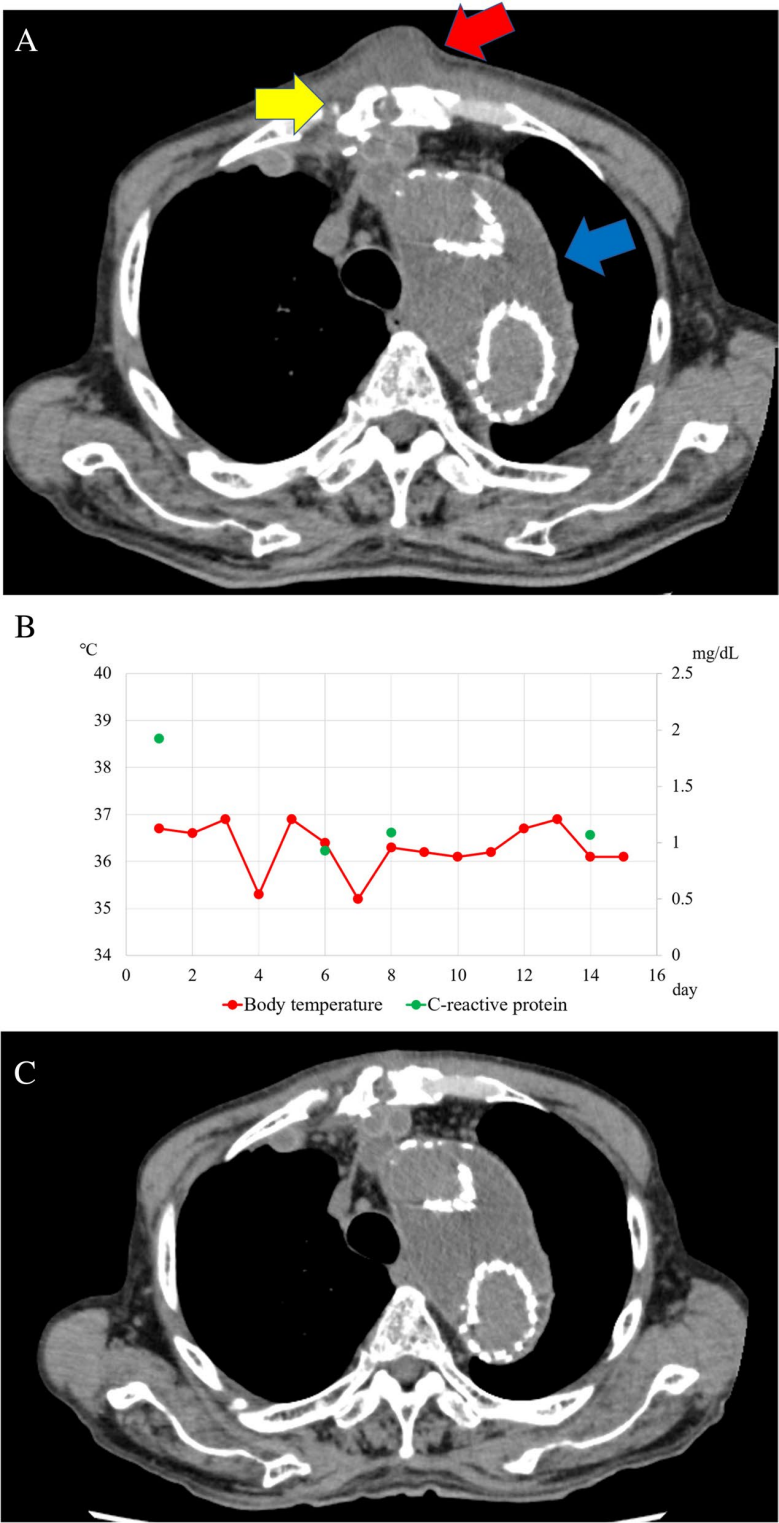
**A** CT findings in case 3. CT revealed a subcutaneous low-density mass (red arrow) sized 20 × 23 mm in the center of the median sternotomy, continuous with a 14-mm-wide area of fluid collection (blue arrow) around the ascending aortic graft. The gap of the closed sternum was also visualized (yellow arrow). **B** Chart for recording case 3 patient body temperature and C-reactive protein level. Shown is the chart of body temperature (red point) and C-reactive protein levels (green point) recorded during hospitalization. No fever was noted during hospitalization, and the C-reactive protein level remained stable without antibiotic therapy. **C** CT findings 1 month after initial treatment with aspiration drainage. The subcutaneous tumor had disappeared

An 84-year-old male with a history of hypertension, chronic subdural hematoma, and cerebral infarction underwent first-stage total arch replacement using a four-branch polyester woven Dacron graft (26 × 9 × 9 × 11-mm J-graft, Japan Lifeline, Tokyo, Japan) with an ET for an aortic arch aneurysm, followed by a second-stage endovascular ET completion procedure. A subcutaneous tumor approximately 2 cm in diameter without reddening appeared in the center of the median sternotomy area at 3 months after the first procedure. CT showed a subcutaneous low-density mass sized 20 × 23 mm, with a continuous 14-mm-wide area of fluid collection around the ascending aortic graft and no extravasation of contrast media observed (Fig. 2A). A physical examination showed a normal body temperature of 36.7°; while blood tests revealed a WBC count of 6240/μL and CRP level of 1.92 mg/dL. Exploratory aspiration of the tumor showed white turbidity, though culture test results were negative. There was no evidence of wound infection or graft infection, including normal body temperature, negative aspiration culture, and no infectious signs in the area of the wound. The tumor was suspected to be a perigraft seroma. Continuous aspiration drainage under negative pressure was performed with a small-bore aspiration catheter (8Fr Argyle^TM^ Fukuroi trocar aspiration kit, Cardinal Health, Dublin, Ohio), inserted in the same manner as in Case 2, and also with use of a J-VAC continuous negative pressure system. During hospitalization, the drainage culture remained negative, and there was no fever. Furthermore, the WBC count and CRP level did not elevate, and the tumor disappeared; thus, the drain tube was removed at 7 days after insertion (Fig. 3C).

Three months after discharge, recurrence of the subcutaneous tumor was noted. A physical examination showed a normal body temperature of 36.7 °C, while blood tests indicated a WBC count of 5580/μL and CRP level of 3.04 mg/dL. We suspected that a perigraft seroma had caused this tumor, and it was treated with continuous drainage in the same manner as before. The WBC count and CRP level remained stable without antibiotic therapy, and the tumor was reduced and the drain tube removed at 7 days after insertion. Thereafter, there was no subcutaneous tumor recurrence, and perigraft fluid collection disappeared after discharge.

## Discussion and conclusions

Spartera et al. reported postoperative perigraft fluid collection areas greater than 3.5 cm in diameter at 1 month after the operation in 19% of cases of aortic reconstruction, with postoperative perigraft fluid collection often noted [1]. However, a subcutaneous tumor due to development of a perigraft seroma after thoracic aortic surgery is an infrequently encountered complication, with only three cases reported thus far in literature [2–4]. In order to effectively treat this subcutaneous tumor, confirmation of diagnosis is important, and when it appears secondary to an anastomotic rupture, pseudoaneurysm, or graft infection, treatment must be performed as soon as possible. The diagnosis is made based on physical examination, blood test, bacterial culture of the tumor, CT, and radiodensity results, as well as other indications. However, Go et al. reported a case with a perigraft seroma that appeared after a Bentall operation, in which artifacts seen in enhanced CT images were considered to be extravasation of contrast media and an anastomotic rupture was mistakenly diagnosed, resulting in a redo sternotomy [3].

As noted in the present cases, confirmation of diagnosis is sometimes difficult. In all three patients, CRP levels were not normal, though none had a fever. Enhanced CT findings in case 1 excluded the possibility of a pseudoaneurysm or anastomotic rupture, whereas the possibility of infection could not be denied. Thus, incisional drainage for the tumor was performed, and then the wound culture was examined and NPWT induced. Even though wound cleansing and NPWT were continued, and wound culture results remained negative, the patient developed a fever 14 days after incisional drainage, and CT revealed perigraft gas formation. It was assumed that the graft infection and mediastinitis were caused by a retrograde infection from the incised subdermal tumor. For cases 2 and 3, in consideration of the risk of such retrograde infection, the tumor was treated with continuous aspiration drainage with negative pressure, not incised drainage, because enhanced CT findings showed no evidence of a pseudoaneurysm, anastomotic rupture, or infection, while blood tests and a physical examination also indicated no evidence of an infection. In these latter two cases, the drainage cultures remained negative, and the tumor and perigraft fluid collection disappeared during the follow-up course.

Generally, treatments used for a perigraft seroma include aspiration of fluid, incision to provide drainage, replacement of the affected graft, and wrapping the affected graft with heterogeneous pericardium, as well as others related to the condition [5, 6]. In a survey reported by Blumenberg et al., the success rate was highest with graft replacement (92%) and lowest with fluid aspiration (70%) [5]. However, in elderly patients with increased risk, resolution may occasionally be achieved by watchful waiting with or without multiple aspiration procedures [5]. The present cases 2 and 3 patients were both elderly and had increased risk associated with a redo sternotomy or graft replacement; thus, fluid aspiration was performed in both. In case 2, the subcutaneous tumor disappeared following a single aspiration treatment, while recurrence was noted in case 3 at 3 months after the initial aspiration. Thus, aspiration was performed again, and no recurrence was seen for up to 3 years. This treatment is considered to be reasonable for elderly patients with high risk.

Serous ultrafiltrate extravasation through the graft wall can eventually result in a perigraft seroma [7], with concomitant anticoagulant therapy, platelet malfunction, hypertension, anemia, diabetes, smoking, and other such factors affecting development [8, 9]. In each of the present cases, antiplatelet or anticoagulant therapy was used because of a history of cerebral infarction or atrial fibrillation, which can lead to formation of a perigraft seroma. Moreover, in all three, cervical branch reconstruction was performed, with the pericardium around the ascending aorta not sutured and closed. Therefore, the bypassed graft to the cervical branches was quite close to the posterior sternum and those made contact at some parts. Postoperative perigraft fluid was able to pass through the gap of the closed sternum and form a subcutaneous tumor. When both perigraft fluid and a subcutaneous tumor are recognized, the subcutaneous tumor should be treated because it might have been caused by a perigraft seroma.

Should a subcutaneous tumor develop after thoracic aortic surgery, differential diagnosis possibilities include anastomotic rupture, pseudoaneurysm, infection, and seroma, as well as others. Confirmation of diagnosis is required for proper treatment. When the possibility of anastomotic rupture or pseudoaneurysm are excluded based on CT findings, incisional drainage may not be effective, as a perigraft seroma can cause a subcutaneous tumor. In such cases, continuous aspiration drainage is a useful option as a diagnostic method and for treatment.

## Abbreviations

CT: Computed tomography
NPWT: Negative pressure wound therapy
ET: Elephant trunk
